# Dexmedetomidine versus ketamine as adjuvants in external oblique intercostal plane block for post thoracotomy pain: a randomised trial

**DOI:** 10.1186/s12871-025-03476-2

**Published:** 2025-11-26

**Authors:** Saad Ahmed Moharam, Mohammed Said ElSharkawy, Ahmed Shehata Abdelhamid, Asmaa Abdelbadie, Neveen A. Kohaf, Mohamed Abd El Rahman Elgaria, Amr Attia, Ahmed Mohamed Elkashef

**Affiliations:** 1 Anesthesiology, Surgical Intensive Care and Pain Medicine Department, Faculty of Medicine, Tanta University, Tanta, 31511 Egypt; 2 Anaesthesia and Intensive care Department, Faculty of Medicine, Port Said University, Port Said, Egypt; 3 Clinical Pharmacy Department, Faculty of Pharmacy, Nahda University, Beni Suef, Egypt; 4 Clinical Pharmacy Department, Faculty of Pharmacy, Al-Azhar University, Cairo, Egypt; 5 Cardiothoracic Surgery Department, Faculty of Medicine, Tanta University, Tanta, Egypt

**Keywords:** Dexmedetomidine, Ketamine, Adjuvant, External oblique intercostal plane block, Thoracotomy, Pain

## Abstract

**Background:**

The external oblique intercostal plane block (EOIPB) is a novel block that targets explicitly the intercostal nerves to provide pain relief, reduce opioid use, and improve postoperative recovery. This study evaluated the effectiveness of dexmedetomidine (Dex) and Ketamine as adjuvants in EOIPB for post-thoracotomy pain (PTP).

**Methods:**

This double-blind, randomised trial involved forty adults undergoing open thoracotomy. Cases were distributed equally into two groups. The Ketamine group received bupivacaine 0.25% with Ketamine, while the Dex group received bupivacaine 0.25% with dexmedetomidine.

**Results:**

Patient-required intraoperative fentanyl consumption was not different between groups. Dex provided superior postoperative analgesia to Ketamine, with significantly lower pain scores at 8 h (median 2 vs. 3, *p* = 0.001) and 12 h (*p* = 0.035). Time to first rescue analgesia was prolonged (14.65 ± 2.25 vs. 10.26 ± 1.73 h, *p* < 0.001), and morphine consumption was notably reduced in the Dex group at both 24 h (5.4 ± 1.57 vs. 6.95 ± 1.43 mg, *p* = 0.003) and 48 h (7.95 ± 1.47 vs. 9.95 ± 1.43 mg, *p* < 0.001). The two groups did not exhibit any substantial variations in patient satisfaction and the incidences of bradycardia, hypotension, and postoperative nausea and vomiting.

**Conclusions:**

As an adjuvant in EOIPB, Dex is superior to Ketamine. It provides a longer duration of analgesia, significantly reduces postoperative opioid consumption, and results in lower pain scores at specific time intervals, without a significant difference in adverse effects.

**Trial registration:**

Registration at clinicaltrials.gov (NCT06331182) Date of registration: 2024-03-17.

## Background

Severe pain is commonly experienced by individuals undergoing open thoracotomy for lung malignancy or other lung procedures [1, 2]. Consequently, this pain prolongs the time it takes to recover after surgery and continues as a persistent condition known as chronic post-thoracotomy pain (PTP) syndrome [3].

High opioid doses can trigger tolerance, hyperalgesia, and side effects like sedation, breathing issues, nausea, vomiting [4]. Thoracic epidural analgesia is often regarded as an effective and reliable method for managing pain following thoracotomy [5], but there are potential side effects associated with its use, including bradycardia, hypotension, nerve injury, nausea and vomiting, and pneumothorax [6].

The external oblique intercostal plane block (EOIPB) is a novel block that intended explicitly the intercostal nerves responsible for transmitting pain signals from the thoracic region [7]. EOIPB is designed to provide pain relief during surgical procedures [8]. One benefit of EOIPB is that it can be performed while the patient is lying on their back, which can simplify the procedure than other blocks [9]. EOIPB has been demonstrated to provide adequate analgesia for various procedures; it effectively reduces opioid consumption and pain scores in subjects had laparoscopic sleeve gastrectomy [9] and laparoscopic cholecystectomy [10].

The use of adjuvants like Ketamine and dexmedetomidine (Dex) combined with bupivacaine in pain management is gaining interest. These adjuvants can enhance the analgesic effects of bupivacaine, decrease the necessity for opioid medications, and improve overall pain control [11].

Ketamine, an N-methyl-D-aspartate (NMDA) receptor antagonist, alleviates pathological pain and opioid tolerance by inhibiting nociceptive stimulation before surgery, countering NMDA receptor activation that enhances acute opioid tolerance [12]. Ketamine is employed as an adjuvant to reduce the total quantity of opioid consumed, delayed the time of the initial rescue analgesia, and reduce the pain score, all without significantly affecting chronic pain and complications [13, 14].

Dex is a medication that activates specifically alpha 2-adrenoceptors, resulting in sedation, anxiety reduction, and pain relief without inducing respiratory depression [15]. As a result, it is commonly used as an adjuvant to increase the duration of peripheral nerve blockade and decrease the need for opioids during and after surgery [16, 17].

Multiple studies have compared Dex and Ketamine as adjuvants to bupivacaine in different blocks [18–20], but this is the first study to compare them in EOIPB.

Given their distinct mechanisms of action, a direct comparison is warranted to identify the optimal adjuvant for this block. We hypothesised that Dex, as an adjuvant to bupivacaine in EOIPB, would provide superior postoperative analgesia than Ketamine, as evidenced by a longer duration of the block, reduced opioid consumption, and lower pain scores in the first 48 h after thoracotomy.

Therefore, this trial’s objective is to compare the effectiveness of Dex and Ketamine when utilised as an adjuvant in EOIPB for managing PTP.

## Methods

Involving 40 patients from both sexes, all aged no less than 18 and with ASA physical status I-III, this double-blind randomised trial took place at Tanta University Hospitals, Egypt, for those undergoing open thoracotomy. The protocol was authorized by the Institutional Ethical Committee at the Faculty of Medicine, Tanta University, Tanta, Egypt (Approval code: 36264PR552/2/24), registered via clinicaltrials.gov (NCT06331182), and ran from March 2024 until November 2024. This study was done in compliance with the Helsinki Declaration. Written consent, informed in nature, was gathered from the subjects.

The exclusion criteria were neurological or cognitive impairment, localised infection at the site of injection, abnormalities in coagulation, allergic reaction to local anesthetics, diabetes mellitus, drug addiction, opioid addiction, pregnancy, severe cardiovascular issues, severe renal and/or hepatic failure, and uncontrolled hypertension.

### Randomisation and blindness

Participants were randomly assigned using computer-generated randomisation numbers using an online randomisation program (http://www.randomizer.org) to produce a random list, and the code of each patient was stored in an opaque sealed envelope. Patients were assigned randomly to two groups using a 1:1 allocation ratio in a parallel manner. The Ketamine group: patients received 29 ml bupivacaine 0.25% plus Ketamine 50 mg diluted in one ml saline 0.9%. The Dex group: patients received 29 ml bupivacaine 0.25% plus Dex 0.5 µg/kg diluted in one ml saline 0.9%.

A clinical pharmacist, not involved in any other aspect of the study, prepared the study solutions in identical 30 ml syringes according to the randomisation list. These syringes were labelled only with the patient study number and were handed to the anesthesiologist performing the block, who was also blinded to the group assignment. The attending anesthesiologist, surgical team, patients, and the researcher responsible for postoperative data collection were all unaware of the group allocation. This process ensured double-blinding throughout the trial.

Medical and surgical history taking, clinical examination, and standard laboratory tests such as CBC, coagulation studies, renal function and liver function were performed preoperatively. Patients were instructed about the Numerical Rating Scale (NRS).

Pulse oximetry, electrocardiogram (ECG), non-invasive blood pressure, a temperature probe, and capnography were standard intraoperative patient monitoring measures. All subjects received 2 mg IV midazolam after cannula placement. GA was induced with propofol 1.5–2.5 mg/kg, fentanyl 1 µg/kg, and cis-atracurium 0.15 mg/kg, followed by intubation, sevoflurane 2% with 50% oxygen, and incremental cis-atracurium 0.03 mg/kg. The patients were placed on mechanical ventilation (MV) to sustain end-tidal CO_2_ levels between 35 and 40 mmHg.

Blocks were administered immediately following the induction of GA and before the skin incision.

### Technique of EOIPB

Utilizing the approach outlined by Elsharkawy et al. [7], participants assumed a supine posture while abducting the ipsilateral arm. The ultrasound (US) transducer was aligned in a parasagittal manner above the sixth rib, positioned medial to the anterior axillary line. Researchers pinpointed the external oblique intercostal plane, which lies above the intercostal muscles between the sixth and seventh ribs. In-plane progression of the needle occurred cephalad, with subsequent hydrodissection conducted using 3 mL saline.

Before the block was performed and at 15-minute intervals until the operation was finished, baseline, and during the procedure, heart rate (HR) and mean arterial blood pressure (MAP) were recorded. If the HR or MAP was elevated by greater than 20% from the baseline (with the exclusion of any other causes other than pain), additional fentanyl loading doses of one µg/kg were administered IV.

After the surgery, anaesthetic administration was stopped, and to reverse muscle relaxation, atropine (0.02 mg/kg) and neostigmine (0.08 mg/kg) were administered. Following this, extubation was administered. Once the patients regain full consciousness, they are promptly sent to the post-anesthesia care unit (PACU).

A standardised pain relief treatment was administered for the period after the surgery. Every six hours, every patient was administered 1 gram of paracetamol as routine pain relief. NRS was evaluated at 0, 4, 8, 12, 18, 24, 36, and 48 h postoperatively. Morphine was administered as a 3 mg bolus for rescue analgesia if the NRS score was greater than three. If the pain persists, the dose can be repeated after 30 min to keep the NRS score lower than four.

The adverse effects were recorded and managed. Hypotension (MAP decreased more than 20% from baseline or < 65 mmHg; treated with IV fluids/ephedrine 5 mg), bradycardia (HR < 50 beats/min; atropine 0.02 mg/kg), respiratory depression (oxygen saturation (SpO_2_) < 95%; supplemental oxygen), postoperative nausea and vomiting (PONV) (ondansetron 1 mg IV), pneumothorax, and local anesthetic systemic toxicity (LAST)were recorded as complications.

The main endpoint was time to the first request of rescue analgesia. The secondary endpoints were intraoperative fentanyl consumption, total morphine consumed within the first 24 and 48 h, pain score, intraoperative hemodynamics, level of patient satisfaction [evaluated using a 5-point Likert scale (1, extremely dissatisfied; 2, unsatisfied; 3, neutral; 4, satisfied; 5, extremely satisfied) [21] and adverse effects.

### Sample size

G*Power 3.1.9.2 (Universitat Kiel, Germany) was utilised. We conducted an unpublished pilot study (five cases in each group), and we discovered that the mean (± SD) of time to the first rescue analgesia was 11.6 ± 0.89 h in the Ketamine group and 12.8 ± 1.09 h in the Dex group, by employing a 1:1 group ratio, a 95% confidence limit, 80% power, and an effect size of 1.20. To overcome dropouts, four cases were added to each group. The outcome was that we recruited 20 subjects for each group.

### Statistical analysis

SPSS v27 (IBM, Armonk, NY, USA) was utilised. Data normality was tested with Shapiro-Wilks and histograms. Parametric data were analysed using unpaired Student’s T-test (mean ± SD), non-parametric with Mann-Whitney (median, IQR), and qualitative with Chi-square/Fisher’s exact (frequency, %). *P* ≤ 0.05 indicated significance.

## Results

**Fig. 1:**
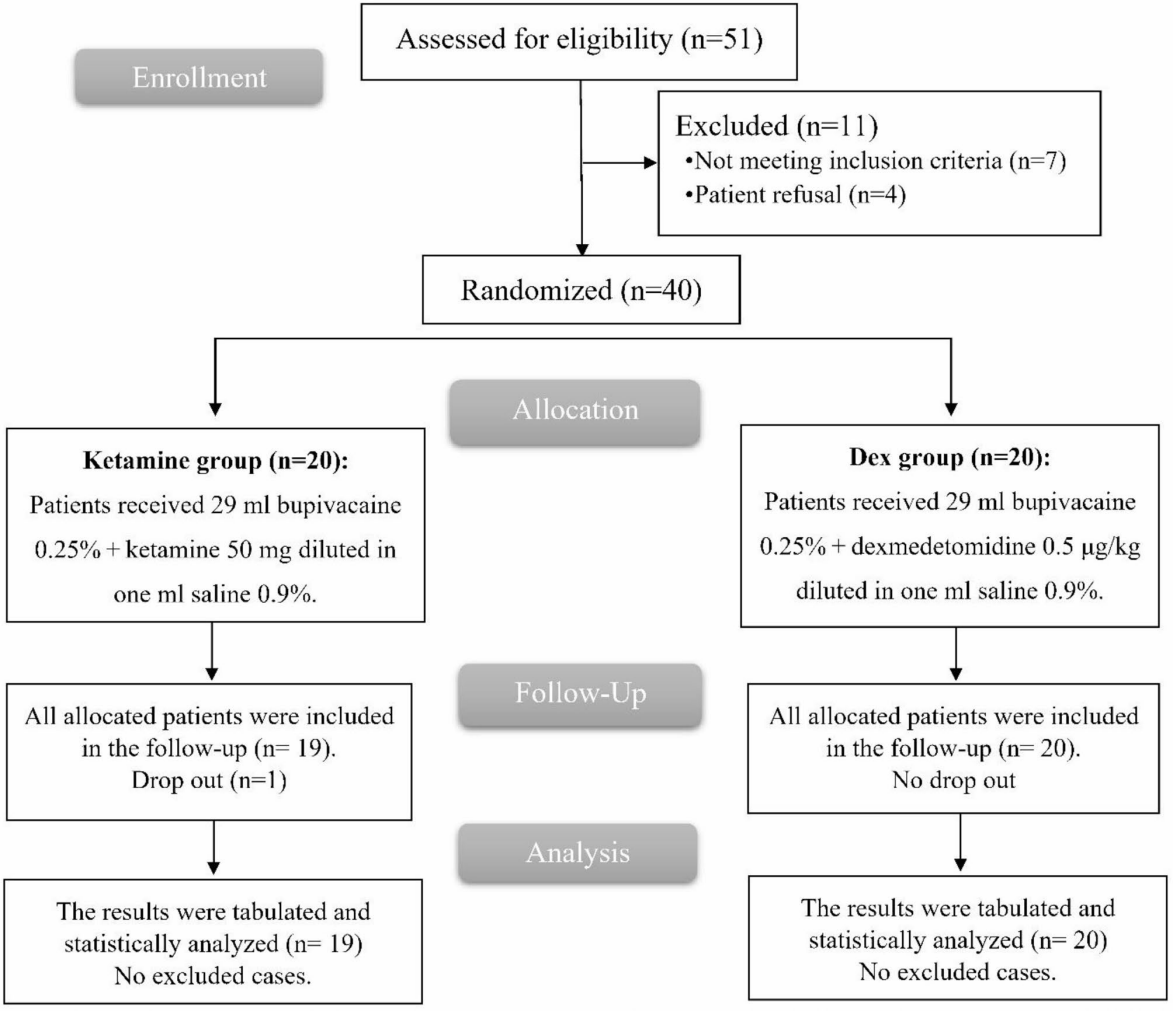
CONSORT flowchart of the enrolled patients

In this trial, 51 patients were evaluated to ascertain their eligibility; seven did not satisfy the eligibility criteria, and four declined to participate. Each of the two groups was randomly allocated twenty patients. Statistical analysis and follow-up of all allocated subjects in the Dex group and 19 patients in the Ketamine group, with one patient withdrawing due to the need for MV following surgery. Fig. 1.

The groups did not exhibit any substantial variations in the demographic data or the duration of the surgery. Table 1.

**Table 1:** Demographic data and duration of surgery of the studied groups

		Ketamine group (*n* = 19)	Dex group (*n* = 20)	*P* value
Age (years)		58.32 ± 12.88	56.9 ± 12.55	0.730
Sex	Male	14 (73.68%)	13 (65%)	0.731
	Female	5 (26.32%)	7 (35%)	
Weight (kg)		78.68 ± 11.13	74.5 ± 8.78	0.199
Height (cm)		168.89 ± 5.84	169.5 ± 5.98	0.751
Body mass index (kg/m^2^)		27.74 ± 4.55	26.04 ± 3.71	0.209
ASA physical status	I	3 (15.79%)	5 (25%)	0.526
	II	11 (57.89%)	8 (40%)	
	III	5 (26.32%)	7 (35%)	
Type of surgery	Lobectomy	8 (42.11%)	7 (35%)	0.890
	Pneumonectomy	3 (15.79%)	3 (15%)	
	Wedge resection	2 (10.53%)	1 (5%)	
	Bullectomy	6 (31.58%)	9 (45%)	
Duration of surgery (min)		165.26 ± 27.76	168.25 ± 22.61	0.714

Data expressed as mean ± SD or frequency (%)

**Fig. 2:**
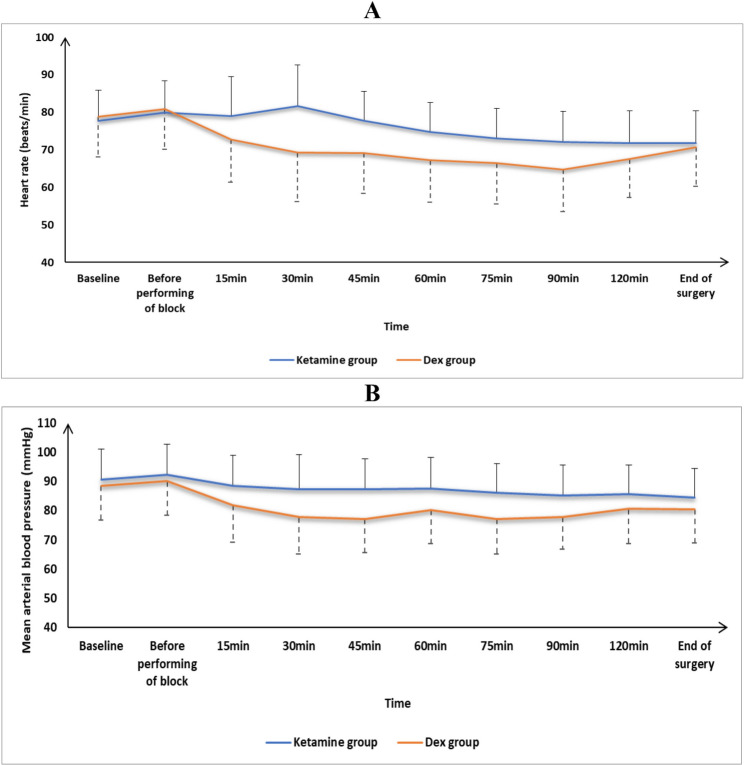
**A** Heart rate and **B** mean arterial blood pressure changes of the studied groups

HR and MAP records were comparable at baseline, before performing the block, 15 min, 120 min, and at the end of surgery between the groups and were markedly reduced at 30 min, 45 min, 60 min, 75 min, and 90 min in the Dex group than in Ketamine group (*P* < 0.05). Fig. 2.

NRS measurements were insignificantly different at 0, 4, 18, 24, 36, and 48 h between the groups and were notably reduced at 8 h and 12 h in the Dex group than in Ketamine group (*P* < 0.05). Table 2.

**Table 2:** Numeric rating scale (NRS) of the studied groups

	Ketamine group (*n* = 19)	Dex group (*n* = 20)	*P* value
0 h	1 (0–1)	1 (0–1)	0.667
4 h	2 (1–2)	1 (1–2)	0.513
8 h	3 (2.5–4)	2 (2–2)	0.001
12 h	3 (2–5)	2 (2–4)	0.035
18 h	4 (4–5)	3 (2–5)	0.065
24 h	4 (3–4)	4 (2.75–5)	0.531
36 h	4 (2–4.5)	3 (2–4.25)	0.647
48 h	3 (2–4)	3 (2.75–4)	0.380

Data expressed as median (IQR)

No substantial variation was observed between groups in patients requiring intraoperative fentanyl or total fentanyl use. However, the Dex group showed longer time to first rescue analgesia (*P* < 0.001) and significantly lower cumulative morphine consumption at 24 and 48 h than in Ketamine group (*P* < 0.05). Table 3.

**Table 3:** Analgesic outcomes of the studied groups

	Ketamine group (*n* = 19)	Dex group (*n* = 20)	*P* value
The patient required intraoperative fentanyl consumption	5 (26.32%)	1 (5%)	0.091
Total intraoperative fentanyl consumption (µg)	0 (0–17.5.5)	0 (0–0)	0.277
Time to first request rescue analgesia (h)	10.26 ± 1.73	14.65 ± 2.25	< 0.001
The total dose of morphine consumed in the first 24 h (mg)	6.95 ± 1.43	5.4 ± 1.57	0.003
The total dose of morphine consumed in the first 48 h (mg)	9.95 ± 1.43	7.95 ± 1.47	< 0.001

Data expressed as mean ± SD, median (IQR) or frequency (%)

Both groups did not exhibit any substantial variations in the levels of patient satisfaction, bradycardia, hypotension, and PONV. No patient in either group experienced respiratory depression or LAST. Table 4.

**Table 4:** Patient satisfaction and side effects of the groups studied

		Ketamine group (*n* = 19)	Dex group (*n* = 20)	*P* value
Patient satisfaction	Extremely satisfied	3 (15.79%)	8 (40%)	0.267
	Satisfied	7 (35%)	7 (35%)	
	Neutral	8 (40%)	5 (25%)	
	Unsatisfied	1 (5%)	0 (0%)	
	Extremely dissatisfied	0 (0%)	0 (0%)	
Adverse effects	Bradycardia	0 (0%)	3 (15%)	0.230
	Hypotension	1 (5.26%)	5 (25%)	0.181
	postoperative nausea and vomiting (PONV)	4 (21.05%)	2 (10%)	0.407
	Respiratory depression	0 (0%)	0 (0%)	---
	Local anesthetic systemic toxicity (LAST)	0 (0%)	0 (0%)	---

Data expressed as frequency (%)

## Discussion

EOIPB is a recently developed regional anaesthetic technique focusing explicitly on the intercostal nerves responsible for transmitting pain signals from the thoracic region [7]. EOIPB involves depositing anesthetic between the external oblique and intercostal muscles, where it spreads to block intercostal nerves and inhibit thoracic pain transmission [10]. So, it is used particularly for surgical procedures involving the lower thorax and chest. It provides pain relief, reduced opioid use, improved postoperative recovery, minimal impact on respiratory function, and reduced risk of complications [8, 22].

The research focuses on the role of Dex and Ketamine as adjuvants in the EOIPB in reducing the pain intensity after thoracotomy.

In our study, the HR and MAP measurements were significantly reduced at 30, 45, 60, 75, and 90 min in the Dex group than in Ketamine group. This could be attributed to systemic absorption of the adjuvants from the nearby intercoastal vessels.

Concurring with our finding, Pandya et al. [23] demonstrated that the Dex group had significantly reduced levels of HR and MAP than the ketamine group when undergoing epidural analgesia for lower limb orthopaedic operations. Similarly, Hashim et al. [24] found that the HR and MAP measured substantially decreased in the Dex than the Ketamine in US-guided (USG) supraclavicular block (SCB).

In contrast, Shaker et al. [19] found that the intraoperative HR and MAP showed insignificant differences between the group receiving Dex and Ketamine for ESPB in modified radical mastectomy at all measurement points. This difference can be ascribed to variations in block type, surgical procedure, and Dex doses, as they used 1 µg/kg. Moreover, Radbin et al. [11] reported that the intraoperative HR and MAP showed a slight difference between Dex and ketamine groups in the epidural group in patients who underwent femur fracture surgery. This difference is due to variations in the block position (L3–4 or L4–5), type of surgery, and different ketamine doses (25 mg). Furthermore, Mohmed et al. [18] found that the intraoperative HR and MAP showed insignificant differences between the Dex and the Ketamine in USG-SCB. This disparity could be attributed to blocks and Dex doses (1 µg/kg).

The results of our study indicated that there was no substantial disparity between the two groups in the amount of fentanyl that patients required for intraoperative use.

This is in line with Mitra et al. [25], who found that intraoperative fentanyl consumption was slightly different between the Dex and the ketamine groups in lumbar spine instrumentation surgery. However, Radbin et al. [11] revealed that the amount of opioids used during surgery was reduced in the Dex than in the Ketamine in patients who underwent femur fracture surgery. This difference may be due to the different types of surgery, different blocks (epidural nerve block), and different doses of Ketamine used (25 mg).

In our study, NRS measurements and the cumulative amount of morphine consumed within the first 24 and 48 h were notably reduced at 8 and 12 h in the Dex than in Ketamine group. Compared to Ketamine, Dex experienced a significantly prolonged time to the initial request for rescue analgesia. Matching Shaker et al. [19], revealed that pain was notably reduced in the patients receiving Dex than received Ketamine for ESPB. Also, they showed that the time of the first rescue analgesic request was notably extended in the patients receiving Dex than those receiving Ketamine for ESPB, and there was a significant decline in the overall intake of opioids in the Dex group than the ketamine group.

This was supported by Radbin et al. [11], who performed femur fracture surgery, and Pandya et al. [23], who conducted lower limb orthopaedic surgeries, revealed that the pain score was considerably reduced in the Dex than in Ketamine group postoperatively.

Concurring with our findings, Mohmed et al. [18] showed that the time at which the first request for pain relief occurred was notably delayed in the patients receiving Dex than those receiving Ketamine in the procedure of USG-SCB. Additionally, the amount of pain relief required overall decreased in the Dex compared to the Ketamine.

Our study revealed a slight difference in patient satisfaction between the Dex and the Ketamine. This was in line with Pandya et al. [23], who stated a slight difference between Dex and ketamine groups.

Our results, consistent with Shaker et al. [19] and Mohmed et al. [18], showed comparable adverse effects between Dex and Ketamine, with slightly higher and easily managed bradycardia and hypotension with a single dose of atropine (0.02 mg/kg) in the Dex group, confirming the clinical safety of Dex 0.5 µg/kg as an EOIPB adjuvant.

Single-centre design and small sample size limit generalizability. Although the initial sample size was based on an unpublished pilot study, post-hoc calculations confirmed adequacy for the primary outcome. However, the study may still be underpowered for specific secondary outcomes, such as intraoperative fentanyl consumption and patient satisfaction, increasing the risk of Type II errors; thus, numerical differences in these measures should be interpreted cautiously. All blocks were performed by a single operator, ensuring consistency but limiting technical success rates’ external validity. While patients and outcome assessors were blinded, unblinding due to the distinct hemodynamic effects of adjuvants (e.g., bradycardia with Dex) cannot be wholly excluded.

Additionally, follow-up was restricted to 48 h, and chronic PTP was not assessed. Moreover, the study lacked a control group. Future multicenter studies with larger cohorts with control group, multiple operators, longer follow-up, and varying block techniques or adjuvant regimens are warranted.

## Conclusions

Dex was more effective than Ketamine as an adjuvant for bupivacaine in EOIPB in postoperative pain management, as shown in the reduction of opioid consumption and pain score and delaying time to first rescue without a significant difference in side effects.

## Data Availability

Data is accessible upon reasonable author request.

## Abbreviations

Dex: Dexmedetomidine
ECG: Electrocardiogram
EOIPB: External oblique intercostal plane block
GA: General anaesthesia
HR: Heart rate
IQR: Interquartile range
IV: Intravenous
LAST: Local anaesthetic systemic toxicity
MAP: Mean arterial blood pressure
NMDA: N-methyl-D-aspartate
NRS: Numerical Rating Scale
PACU: Post-anaesthesia care unit
PONV: Postoperative nausea and vomiting
PTP: Post-thoracotomy pain
SD: Standard deviation
